# Septic shock and vasopressor requirement is associated with lower vitamin D levels in critically ill children

**DOI:** 10.1186/cc10390

**Published:** 2011-10-27

**Authors:** K Madden, HA Feldman, E Smith, CM Gordon, S Keisling, R Sullivan, BW Hollis, AA Agan, AG Randolph

**Affiliations:** 1 Department of Anesthesia, Children's Hospital Boston, MA, USA; 2 Department of Anaesthesia, Harvard Medical School, Boston, MA, USA; 3 Division of Endocrinology, Children's Hospital Boston, MA, USA; 4 The Clinical Research Program, Children's Hospital Boston, MA, USA; 5 Department of Pediatrics, Harvard Medical School, Boston, MA, USA; 6 Division of Adolescent and Young Adult Medicine, Children's Hospital Boston, MA, USA; 7 Department of Pediatrics, Medical University of South Carolina, Charleston, SC, USA

## Introduction

Vitamin D plays an important role in immune and cardiovascular function. There is evidence that low 25-hydroxyvitamin D (25(OH)D) levels are associated with an increased risk of life-threatening infections [1,2]. Our objective was to determine the prevalence of 25(OH)D deficiency (<20 ng/ml) in critically ill children and to identify any association with illness severity and infection.

## Methods

From November 2009 to November 2010, we collected blood samples and clinical data on children (<21 years old) near the time of admission to the pediatric ICU, excluding those admitted for short-term monitoring. We measured plasma 25(OH)D concentrations in plasma using Diasorin radioimmunoassay on all subjects. Vasopressor requirement was measured using the cardiovascular component of the Sequential Organ Failure Assessment (CV-SOFA) score.

## Results

Among 511/818 (62.5%) eligible children, 40.1% were 25(OH)D deficient (median level 22.5 ng/ml (IQR = 16.4, 31.3)). Children with a confirmed (*n *= 144, 28.2%) or suspected (*n *= 94, 18.1%) diagnosis of infection on admission did not have lower 25(OH)D levels overall, except for those presenting in severe septic shock (*n *= 51, median = 19.2 ng/ml, IQR = 12.6, 24.8; *P *= 0.0008). In the multivariate analysis, older age and nonwhite race were associated with vitamin D deficiency while summer season, vitamin D supplementation and formula intake were strongly protective. Patients with higher pediatric ICU admission day illness severity by PRISM-III score quartiles had lower vitamin D levels (OR = 1.19 per 5 ng/ml decrease in 25(OH)D, 95% CI = 1.10, 1.28, *P *< 0.0001) after adjusting for risk factors. When septic shock was added to this model, there was no effect on the association between 25(OH)D level and PRISM-III quartile (OR = 1.18 (95% CI = 1.09, 1.27, *P *< 0.0001)). There was also an inverse association between 25(OH)D level and maximal vasopressor use as measured by the CV-SOFA score in a multinomial regression model (OR = 1.13, 95% CI = 1.01, 1.27, *P *= 0.03). Including septic shock in the multivariable model did not affect the effect of vitamin D level (OR = 1.16, 95% CI = 1.02, 1.31, *P *= 0.02)) on CV-SOFA score.

## Conclusion

The overall prevalence of vitamin D deficiency in critically ill children is high, and patients with severe septic shock had significantly lower vitamin D levels than the general population. This association between vitamin D and septic shock may be due to the cardiovascular effects of vitamin D or to increased severity of infection with diminished 25(OH)D levels. These results suggest a role for the vitamin D axis in sepsis and hemodynamic instability that deserves further investigation.
